# Can Dietary Intake of Vitamin C-Oriented Foods Reduce the Risk of Osteoporosis, Fracture, and BMD Loss? Systematic Review With Meta-Analyses of Recent Studies

**DOI:** 10.3389/fendo.2019.00844

**Published:** 2020-02-03

**Authors:** Ling-Feng Zeng, Ming-Hui Luo, Gui-Hong Liang, Wei-Yi Yang, Xiao Xiao, Xu Wei, Jie Yu, Da Guo, Hong-Yun Chen, Jian-Ke Pan, He-Tao Huang, Qiang Liu, Zi-Tong Guan, Yan-Hong Han, Di Zhao, Jin-Long Zhao, Sen-Rong Hou, Ming Wu, Jiong-Tong Lin, Jia-Hui Li, Wei-Xiong Liang, Ai-Hua Ou, Qi Wang, Zi-Ping Li, Jun Liu

**Affiliations:** ^1^The 2nd Affiliated Hospital of Guangzhou University of Chinese Medicine (Guangdong Provincial Hospital of Chinese Medicine), Guangzhou, China; ^2^Bone and Joint Research Team of Degeneration and Injury, Guangdong Provincial Academy of Chinese Medical Sciences, Guangzhou, China; ^3^The Second Clinical College of Guangzhou University of Chinese Medicine, Guangzhou, China; ^4^Wangjing Hospital of China Academy of Chinese Medical Sciences, Beijing, China; ^5^World Federation of Chinese Medicine Societies, Beijing, China

**Keywords:** BMD loss, dietary intake, osteoporosis or fracture, risk reduction, vitamin C-oriented foods, meta-analysis

## Abstract

**Background:** Several epidemiological studies have been performed to evaluate the association of dietary intake of vitamin C-oriented foods (DIVCF) with risk of fracture and bone mineral density (BMD) loss, but the results remain controversial. Therefore, we conducted a systematic meta-analysis to assess this correlation.

**Methods:** We searched EmBase, PubMed, Web of Science, and the Chinese database CNKI for relevant articles published up to August 2019. Pooled relative risks (RRs) with 95% confidence intervals (CIs) were calculated using the random- or fixed-effects model. Discrepancies were resolved by consultation with a third expert.

**Results:** A total of 13 eligible articles (including 17 studies) with 19,484 subjects were identified for the present meta-analysis. The pooled RR of hip fracture for the highest vs. lowest category was 0.66 (95% CI, 0.47–0.94) for DIVCF, i.e., people with a greater frequency of Vitamin C uptake had a 34% (95% CI, 6%−53%) lower prevalence of hip fracture. In subgroup analyses stratified by study design, gender, and age, the negative associations were statistically significant. Furthermore, the statistical analysis of the association between DIVCF and risk of osteoporosis (RR, 0.66; 95% CI, 0.48–0.92), BMD at the lumbar spine (pooled *r*, 0.15; 95% CI, 0.09–0.23), and BMD at the femoral neck (pooled *r*, 0.20; 95% CI, 0.11–0.34) showed beneficial effects of DIVCF.

**Conclusion:** Our meta-analysis indicates that DIVCF is negatively associated with the risk of hip fracture, osteoporosis, and BMD loss, suggesting that DIVCF decreases the risk of hip fracture, osteoporosis, and BMD loss.

## Background

Osteoporosis is a systemic skeletal disease, which is common in postmenopausal women and is characterized by an increased risk of bone fragility and a decrease in bone mass (1). Bone homeostasis requires a balance between bone-forming osteoblasts and bone-resorbing osteoclasts (2). When this balance is impaired, normal bone remodeling cannot keep bone mass stable, leading to osteopenia and osteoporosis (3). Traditionally, dual X-ray absorptiometry (DEXA), which measures bone mineral density (BMD), is routinely used to assess the risk of fracture in osteoporosis patients. According to the World Health Organization, osteoporosis is one of the most common disorders, and 30–50% of all women in the world suffer from fractures due to osteoporosis throughout their lives (4).

Increasing evidence demonstrates that osteoporosis is affected by genetic factors, low body mass (weight), and lifestyle factors, such as coffee intake, alcohol consumption, soft drink intake, and dietary intake of vitamin C-oriented foods (DIVCF) (5). Diet and physical activity, the two key determinants of body weight, might influence osteoporosis directly. Vitamin C-oriented foods comprise one of the most important components of the diet and have been reported to exert many potential health benefits, because they are rich in vitamins, fiber, phytochemicals, and minerals (6). Several studies have found that DIVCF significantly lowers the risk of breast cancer, hypertension, metabolic syndrome, type 2 diabetes mellitus, depression, inflammatory bowel disease, and all-cause mortality (7, 8). Antioxidants and anti-inflammatory components from such diets are hypothesized to play a vital role in the protective effects against osteoporosis (9).

Although a series of epidemiological studies have been performed to evaluate the association between DIVCF and the risk of osteoporosis (10–22), the results remain controversial. A negative association between DIVCF and the risk of hip fractures was observed in two studies (11, 16), whereas no association was shown in other studies (10, 12–15). The purpose of the present systematic meta-analysis was to assess the correlation between DIVCF and the risk of osteoporosis, hip fracture, and BMD loss.

## Methods

The PRISMA (Preferred Reporting Items for Systematic Reviews and Meta-Analyses) guidelines and others were followed in the current analysis (23, 24).

### Literature Search Strategy

**Figure 1:**
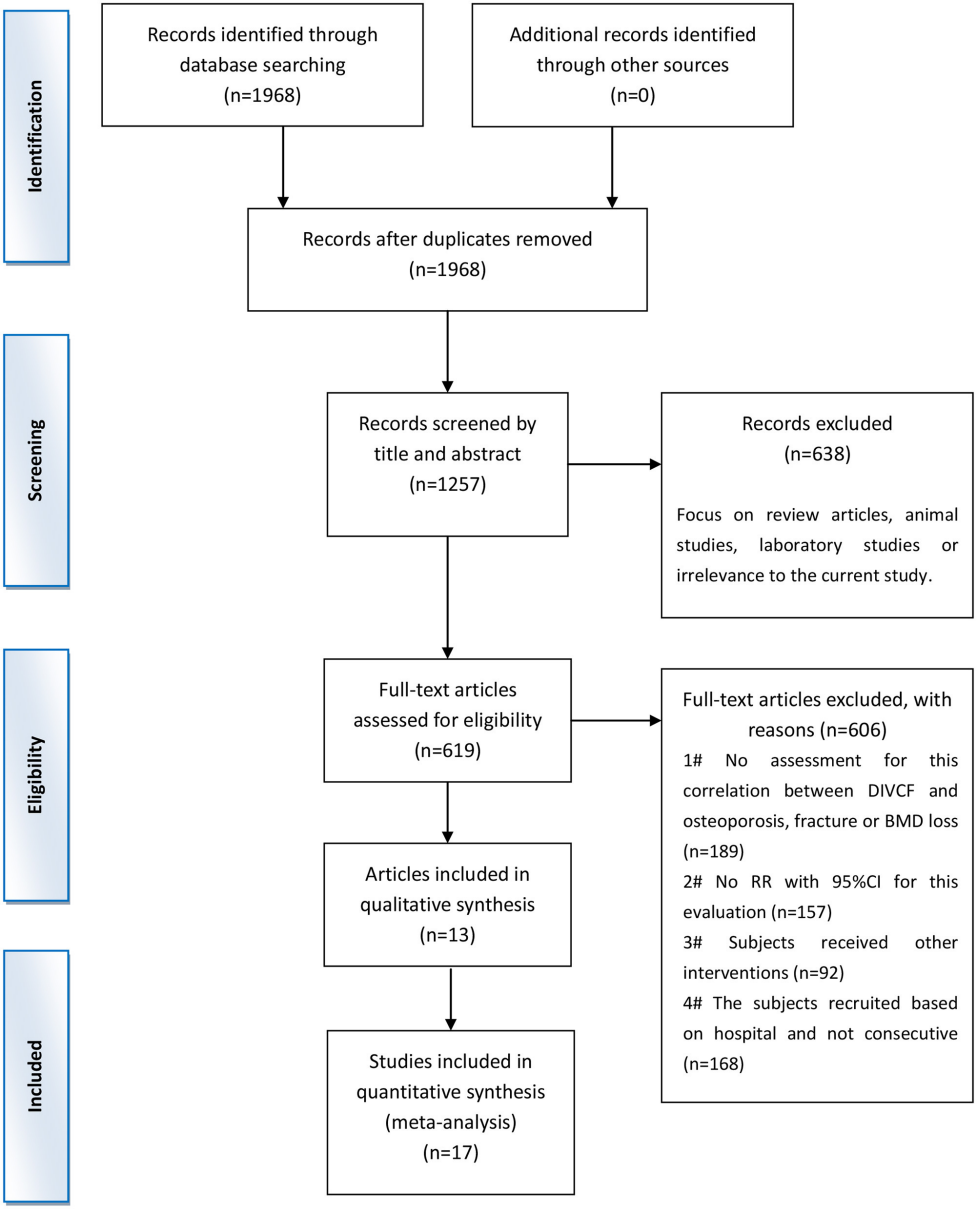
Flow diagram of the trial selection process. DIVCF, dietary intake of vitamin C-oriented foods; BMD, bone mineral density.

A systemic search was performed for potential articles in four databases, i.e., EmBase, PubMed, Web of Science, and the Chinese database CNKI, published in or before August 2019. We used the search terms “Vitamin C,” “ascorbic acid,” “ascorbate,” and “acid ascorbic” in combination with “osteoporosis” or “fracture.” The reference lists from the articles included were manually screened for undetected relevant studies. The final results of the literature search were updated on August 31, 2019. The detailed steps of the literature search are shown in Figure 1.

### Inclusion and Exclusion Criteria

Articles were included in this review if they met the following criteria: (1) observational study published as an original study; (2) exposure of interest was defined as DIVCF, i.e., dietary intake of foods that are rich in Vitamin C; (3) outcome of interest was defined as osteoporosis, fracture, and/or BMD loss in the subjects; and (4) study included estimates of relative risks (RRs), comparing the highest DIVCF category with the lowest, with corresponding 95% confidence intervals (CIs) for the associations or other information that could be used for inference. Furthermore, the following exclusion criteria applied: (1) studies describing animal experiments, review papers, and mechanistic studies; (2) studies lacking specified data of osteoporosis in relation to low bone mass; and (3) articles that only contained an abstract. All identified articles were reviewed independently by two researchers, and discrepancies were discussed with and resolved by a third expert.

### Data Extraction and Quality Assessment

Two researchers extracted data independently, following the standardized norms for literature collection. Any discrepancies were resolved by consulting with a third expert. The following information was extracted from the articles: name of the first author, publication year, country where the study was conducted, baseline age, gender, study design, sample size and number of cases, assessment approach of DIVCF, assessment approach of osteoporosis, the measurement location of osteoporosis, and adjusted RR with 95% CI of osteoporosis for DIVCF consumption. The Newcastle–Ottawa Scale (NOS) was used to assess the quality of case–control and cohort studies (25). The Agency for Healthcare Research and Quality guidelines were applied to assess the quality of cross-sectional studies (26).

### Statistical Analysis

The risk of osteoporosis, fracture, and BMD loss was estimated, comparing the highest DIVCF category to the lowest. Pooled RRs with 95% CIs were calculated using the random-effects model (REM) if moderate or high heterogeneity (*I*^2^ ≥ 50%) was observed; otherwise, the fixed-effects model (FEM) was used. The *I*-squared (*I*^2^) statistic and chi-square test were adopted to explore the possible heterogeneity of the studies. Potential publication bias was inspected by funnel plots. Subgroup analyses were performed by study type, gender, and age to assess potential influencing factors. All statistical analyses were performed using the statistical software package STATA SE, version 14.1 (Stata Corp, College Station, TX, USA).

### Patient and Public Involvement Statement

The study is a systemic literature review that did not involve experimental participants or human subjects, and we did not collect any private data or sensitive information during the review process.

## Results

### Results of the Literature Search

As shown in Figure 1, a total of 1,968 articles were identified in the database search; 1,349 articles were excluded because of the following reasons: 711 articles were duplications, and 638 articles were excluded due to their nature (e.g., reviews, animal studies, or laboratory studies). Of the remaining 619 articles, 189 did not assess the association between DIVCF and the risk of osteoporosis, fracture, or BMD loss; 157 studies did not report the RR (with 95% CI) for the relationship between DIVCF and osteoporosis risk; 92 articles were excluded for other reasons; and 168 studies were based on a hospital-based sample (not on a population-based sample). As a result, 13 published articles (with 17 studies, i.e., 4 cohort, 11 case–control, and 2 cross-sectional studies) were identified as eligible for the present meta-analysis (10–22) (Figure 1).

### Study Characteristics and Quality Assessment

The basic characteristics for DIVCF with risk of osteoporosis are shown in Table 1. In our study quality assessment, the studies received a quality score of ≥5, indicating that the methodological quality of the studies was generally good.

**Table 1:** Characteristics of the studies included in the Meta-analysis.

**References (sources)**	**Study design**	**Gender/Age (y)**	**No. of cases (study size)**	**DIVCF assessment**	**Duration time (y)**	**Outcome**	**Study quality**	**Adjusted factors**
Finck et al. (10) (Male subjects), UK	Cohort	M, 39–79	112/1,842 124/2,334 78/1,808	7-d Food record	12.6	Hip fracture Spine fracture	7	Age, family history of osteoporosis, BMI, smoking, physical activity, steroid medication, energy, Ca intake, Ca and vitamin D supplemental status, HRT
Finck et al. (10) (Female subjects), UK	Cohort	F, 39–79	339/2,525	7-d Food record	12.6	Hip fracture Spine fracture	7	Age, family history of osteoporosis, BMI, smoking, physical activity, steroid medication, energy, Ca intake, Ca and vitamin D supplemental status, HRT
Sun et al. (11) China	Case control	F/M, 42–79	726/1452	Food Frequency Questionnaire	NA	Hip fracture	5	Age, sex, drugs, BMI, education, occupation, income, family history of fracture, smoking, alcohol, Ca and multi-vitamin supplement, physical activity, energy intake
Sahni et al. (12) USA	Cohort	F/M, 70–80	100/958	Food Frequency Questionnaire	15	Hip fracture	6	Age, sex, energy intake, estrogen use, BMI, multi-vitamin use, height
Zhang et al. (13) USA	Case control	F/M, ≥50	1215/2,564	Food Frequency Questionnaire	NA	Hip fracture	8	Age, sex, BMI, physical activity, energy, Ca, vitamin D, protein, caffeine, alcohol intake
Michaelsson et al. (14) Sweden	Case control	F, 40–75	247/1,140	Food Frequency Questionnaire	NA	Hip fracture	7	Diabetes, fracture history, HRT, smoking, physical activity, BMI, energy intake
Nieves et al. (15) USA	Case control	F, 50–103	161/328	Food Frequency Questionnaire	NA	Hip fracture	6	BMI, estrogen use, chronic disease/age and hospital matching
Sun et al. (16) China	Case control	F/M, 40–70	725/1,450	Food Frequency Questionnaire	5	Hip fracture	5	Age, sex, drugs, BMI, educational level, occupation, household income, family history of fracture, smoking, alcohol drinking, calcium supplement use, multivitamin supplement use, physical activity, daily energy intake, and selected dietary nutrients intakes (protein, calcium, and phosphorous:energy-adjusted)
Kim and Lee (17) South Korea	Cross sectional	F/M, ≥50	1212/3,047	24-h recall	NA	Osteoporosis (<-2·5 T-score/LS-FN-TH), BMD	8	Age, sex, income, education, smoking, HRT, survey year, energy intake, Ca intake, blood vitamin D level
Sugiura et al. (18) Japan	Cohort	F, 30–70	17/187	Food Frequency Questionnaire	4	Osteoporosis (T-sore exceeded 70 %/FA)	6	Age, weight, height, years since menopause, current tobacco use, alcohol intake, exercise habit, supplement use, energy intake, intake of Ca, Mg, K, vitamin D
Zhang et al. (19) China	Case control	F, 58.1 ± 6.7	60/159	Food Frequency Questionnaire	NA	Osteoporosis (<-2·5 T-score/LS-FN-TH), BMD	6	Age, sex, BMI, physical activity, energy, Ca, vitamin D, protein, caffeine, alcohol intake
Yang and Kim (20) South Korea	Cross-sectional	M, 50–79	189/2,305	24-h recall	3	BMD	7	Age, weight, education, alcohol intake, exercise, vitamin D, parathyroid hormone
Park et al. (21) South Korea	Case control	F, 50–70	72/144	Food Frequency Questionnaire	NA	Osteoporosis (<-2.5 T-score/LS-FN-FT), BMD	5	Energy intake, age, BMI, HRT/ age-matching
Macdonald et al. (22) UK	Cohort	F, 45–55	5/891	Food Frequency Questionnaire	5	BMD	7	Age, weight, weight change, height, smoking, physical activity, socioeconomic status

*DIVCF, dietary intake of vitamin C-oriented foods; F, female; M, male; NA, Not application*.

### Quantitative Synthesis

#### Meta-Analysis of DIVCF and the Risk of Hip Fracture

**Figure 2:**
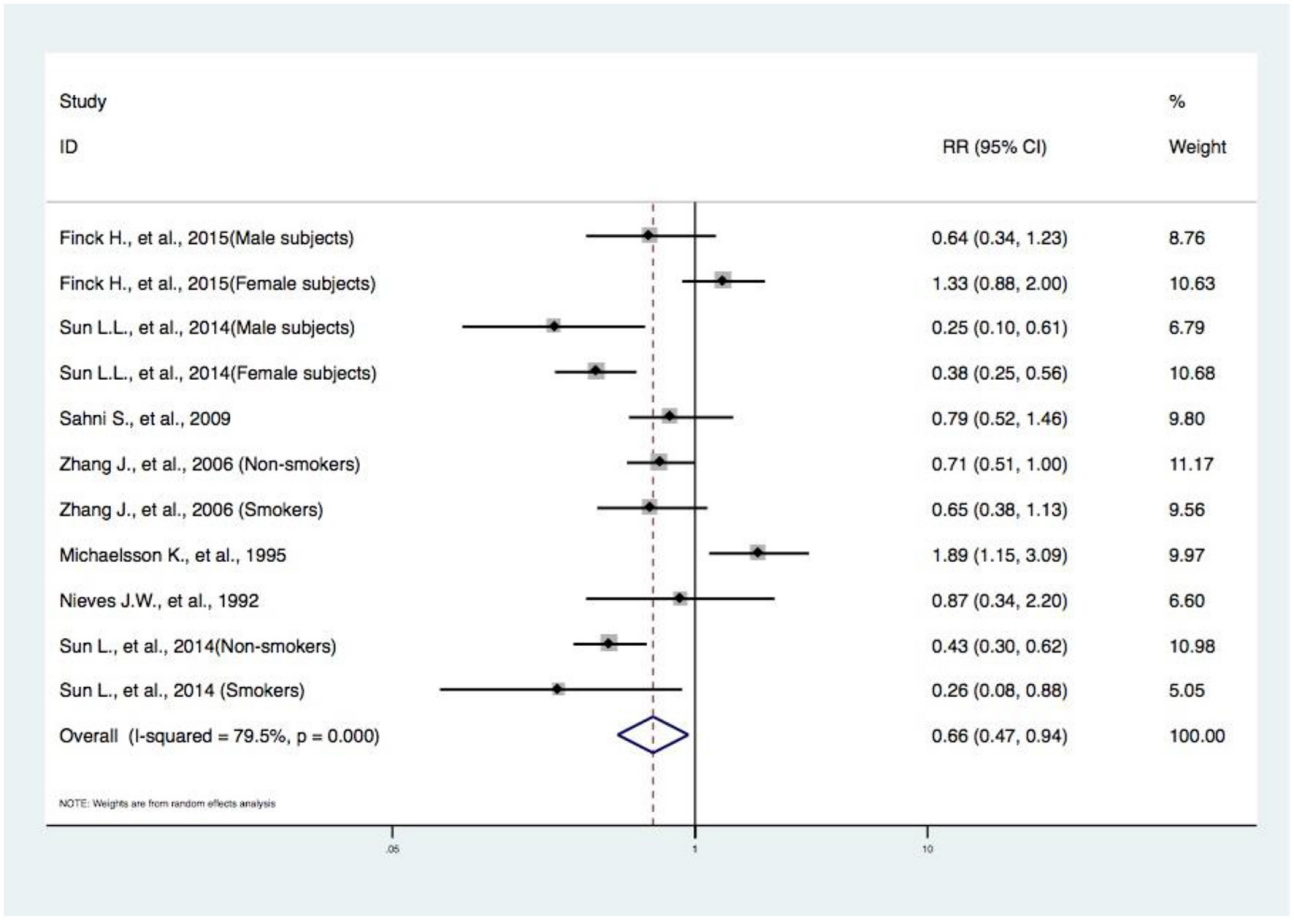
Forest plot of meta-analysis of DIVCF and the risk of hip fracture. The size of the boxes is proportional to the weight assigned to each study, and horizontal lines represent the 95% confidence interval. DIVCF, dietary intake of vitamin C-oriented foods.

**Figure 3:**
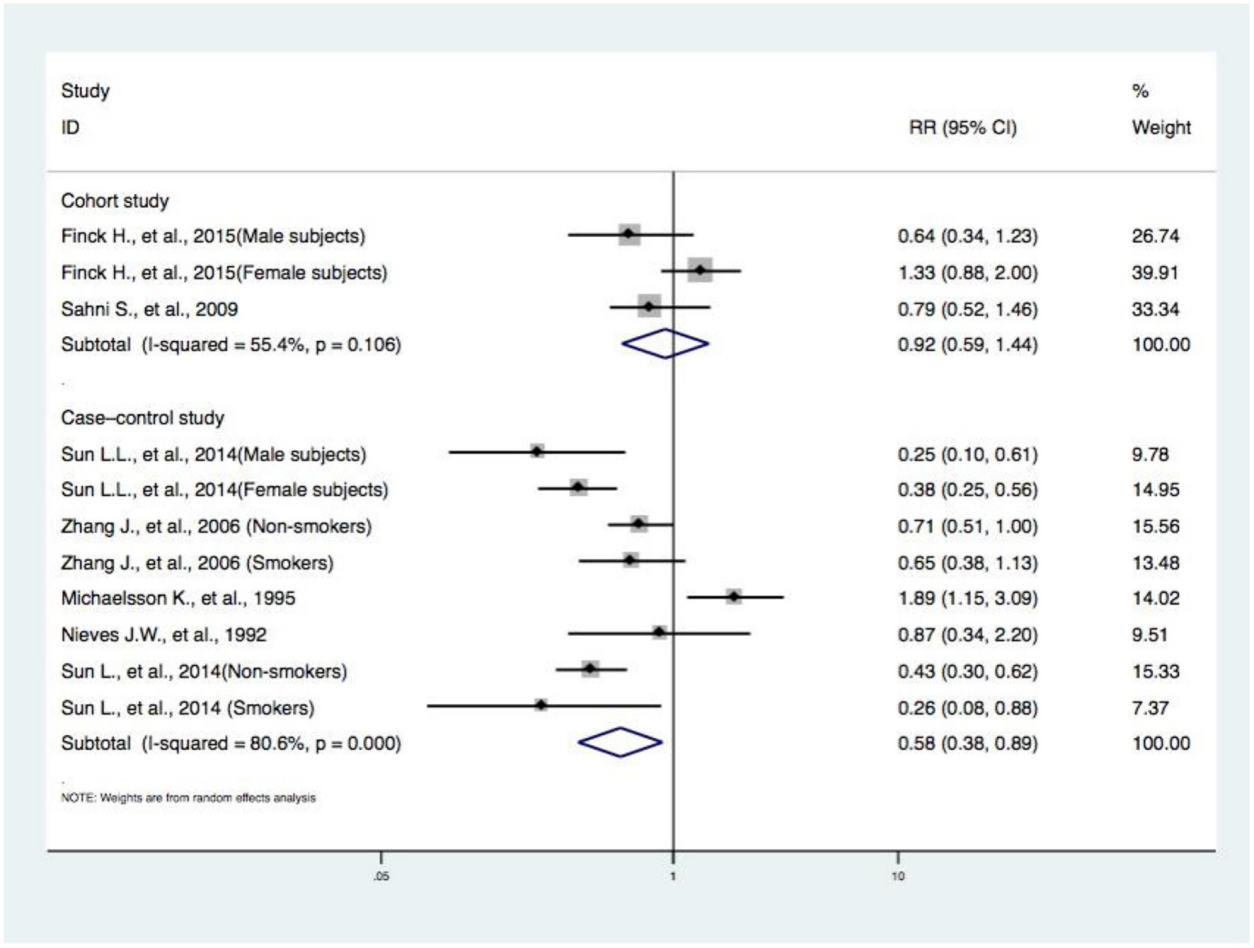
Subgroup analysis of study design concerning DIVCF and the risk of hip fracture. The size of the boxes is proportional to the weight assigned to each study, and horizontal lines represent the 95% confidence interval. DIVCF, dietary intake of vitamin C-oriented foods.

**Figure 4:**
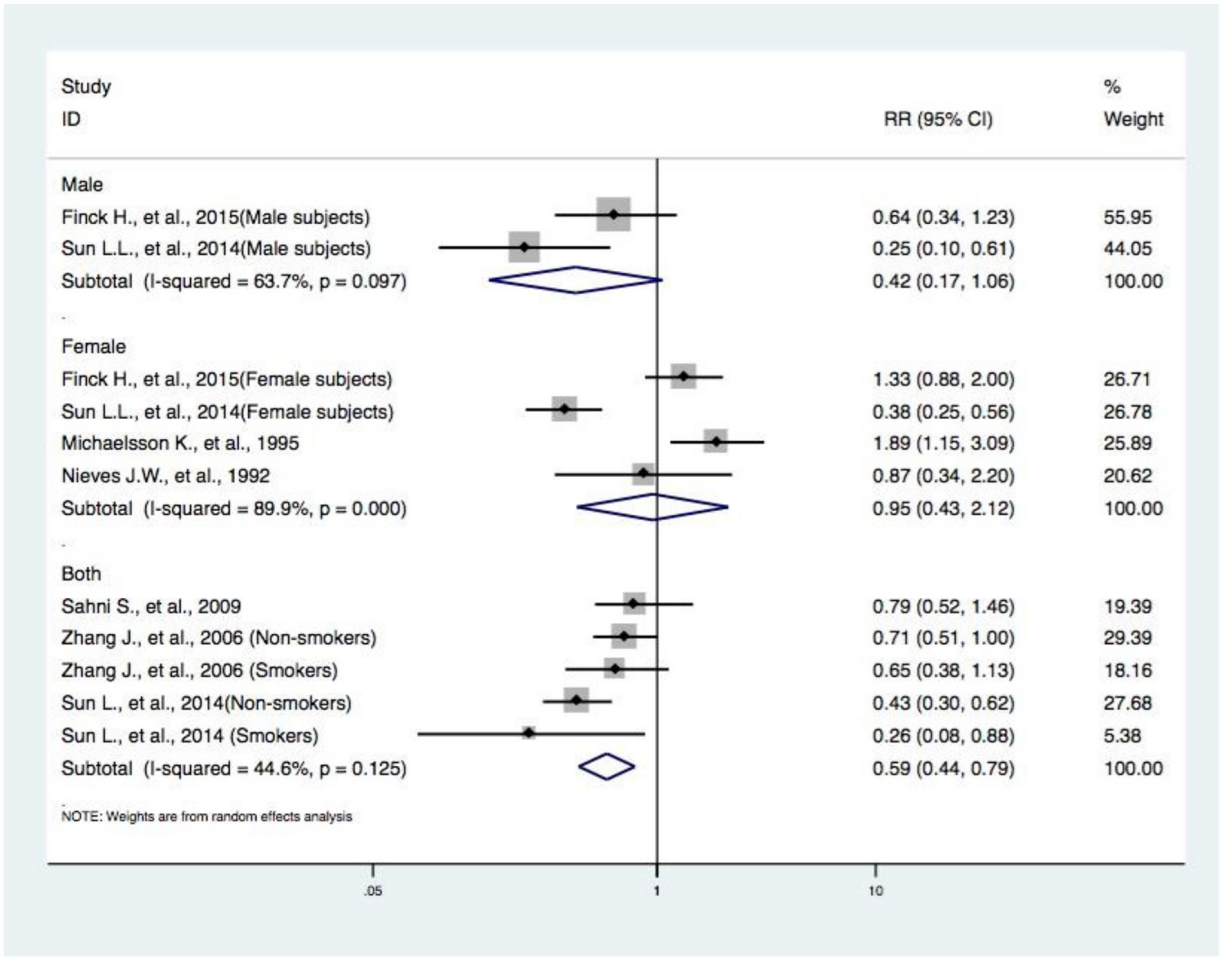
Subgroup analysis of gender concerning DIVCF and the risk of hip fracture. The size of the boxes is proportional to the weight assigned to each study, and horizontal lines represent the 95% confidence interval. DIVCF, dietary intake of vitamin C-oriented foods.

**Figure 5:**
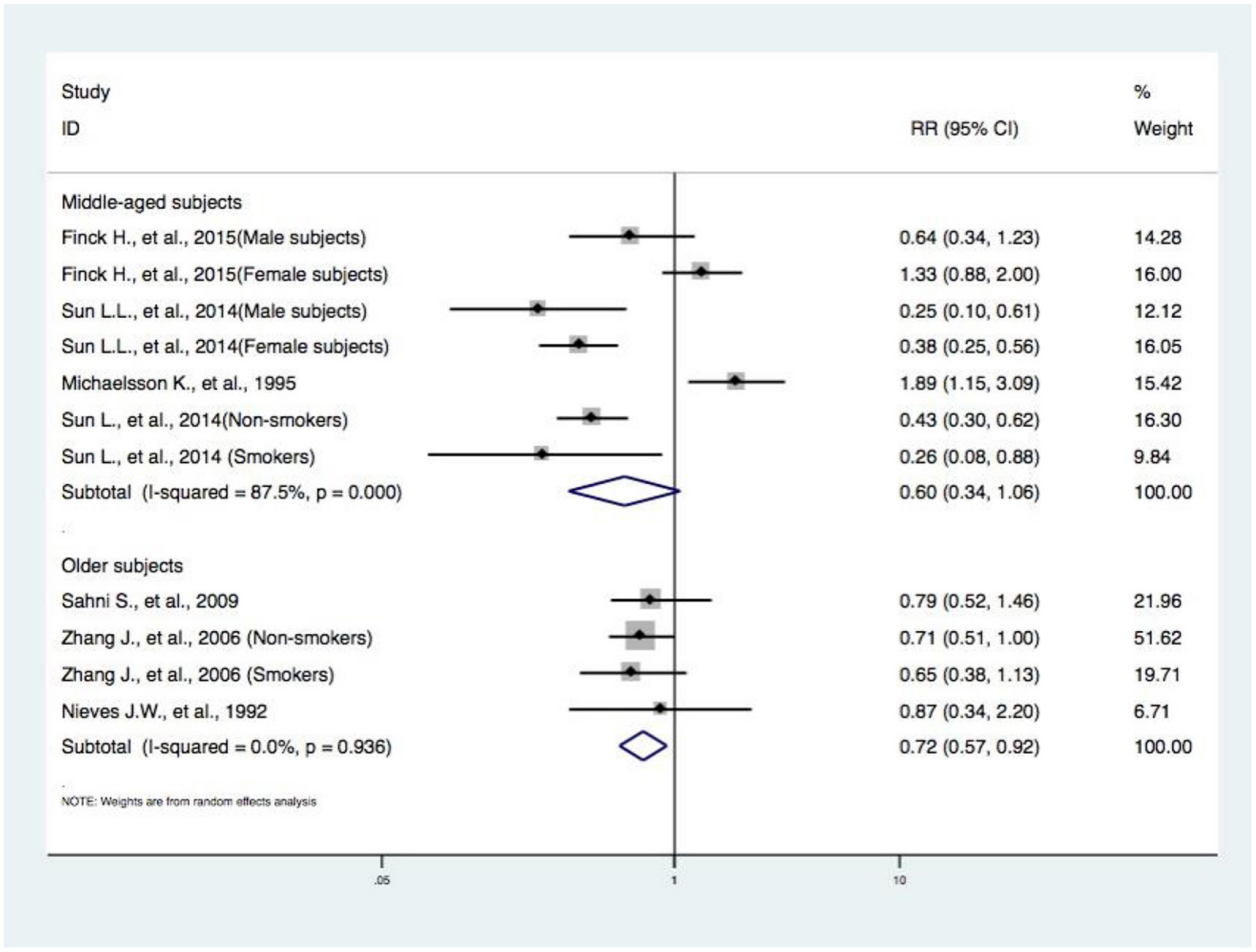
Subgroup analysis of age concerning DIVCF and the risk of hip fracture. The size of the boxes is proportional to the weight assigned to each study, and horizontal lines represent the 95% confidence interval. DIVCF, dietary intake of vitamin C-oriented foods.

Eleven studies from seven articles (10–16) reported the events of hip fracture, and our random-effects meta-analysis showed that there is a significant difference between subjects in the highest category of DIVCF and the lowest. The RR for hip fracture events between the two groups was 0.66 (95% CI, 0.47–0.94) (Figure 2). These results indicate that the people with a higher consumption of Vitamin C-oriented foods had a 34% (95% CI, 6%−53%) lower risk of hip fracture. Because of the high heterogeneity among the abovementioned studies (*I*^2^ = 79.5%; *P* = 0.000), we conducted subgroup analyses based on study type (i.e., cohort and case–control studies), gender, and age (middle-aged subjects, older subjects) (Figures 3–5).

#### Subgroup Analyses by Study Design Concerning DIVCF and the Risk of Hip Fracture

Regarding the studies assessing the correlation between DIVCF and risk of hip fracture, two articles (including four case–control studies) (11, 16) reported that higher DIVCF could lower the risk of hip fracture, while other case–control studies (13–15) failed to reflect such a relationship. Also, two articles (including three cohort studies) (10, 12) failed to identify such a positive association. The RR for DIVCF and risk of hip fracture was much lower in the case–control studies (RR, 0.58; 95% CI, 0.38–0.89), and no significant results were found in the cohort studies (RR, 0.92; 95% CI, 0.59–1.44) (Figure 3).

#### Subgroup Analyses by Gender Concerning DIVCF and the Risk of Hip Fracture

Seven articles (with 11 studies) (10–16) reported the effects of gender in the association between DIVCF and risk of hip fracture. A reduced risk of hip fracture was found for the pooled results of three articles (with five studies) (12, 13, 16) on both male and female subjects (RR, 0.59; 95% CI, 0.4–0.79). However, no positive effects were observed in the synthesized data of two articles (with two studies) (10, 11) on male subjects (RR, 0.42; 95% CI, 0.17–1.06) and four articles (with four studies) (10, 11, 14, 15) on female subjects (RR, 0.95; 95% CI, 0.43–2.12) (Figure 4).

#### Subgroup Analyses by Age Concerning DIVCF and the Risk of Hip Fracture

The RR of hip fracture, from three articles (with four studies) (12, 13, 15) on older subjects (RR, 0.72; 95% CI, 0.57–0.92), is lower among subjects in the highest category of DIVCF. No statistically significant correlations were observed in the analysis of four articles (with seven studies) (10, 11, 14, 16) on middle-aged subjects (RR, 0.60; 95% CI, 0.34–1.06) (Figure 5).

### Publication Bias Analysis

Funnel plots were used to evaluate the potential publication bias, with RRs generated from the articles concerning DIVCF and the risk of hip fracture. In the absence of publication bias, most of the formulated points are symmetrically placed around the vertical line given by the pooled RRs. No evidence of publication bias was found in the evaluation of DIVCF and the risk of hip fracture (Supplementary Figure 1).

### Meta-Analysis of DIVCF and the Risk of Osteoporosis

**Figure 6:**
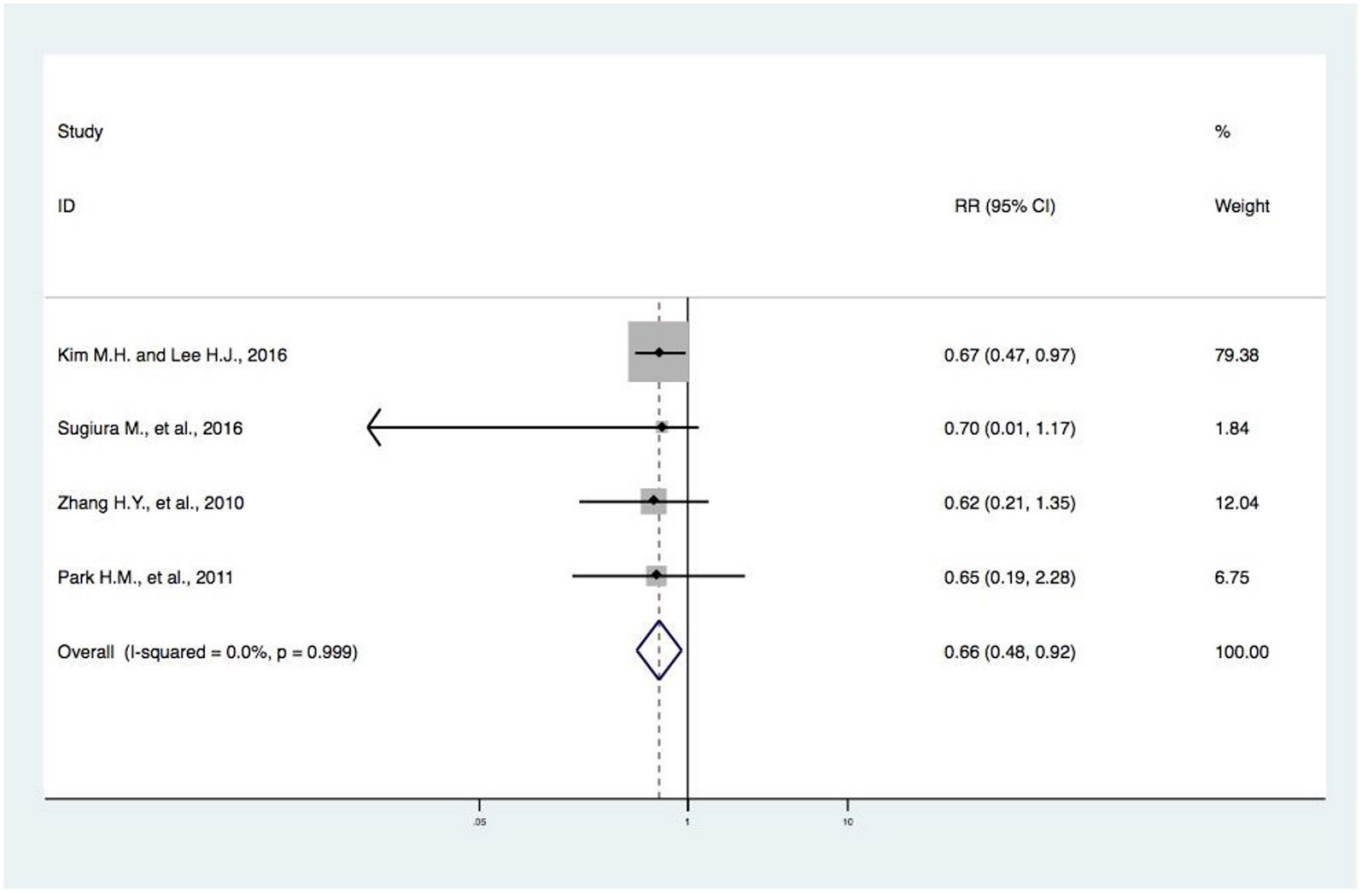
Forest plot of meta-analysis of DIVCF and the risk of osteoporosis. The size of the boxes is proportional to the weight assigned to each study, and horizontal lines represent the 95% confidence interval. DIVCF, dietary intake of vitamin C-oriented foods.

Four studies (17–19, 21) reported on DIVCF and the risk of osteoporosis. One study (17) found a negative association between DIVCF and risk of osteoporosis (RR, 0.67; 95% CI, 0.47–0.97), while no significant association was found in the other three studies (18–20). The evidence synthesis for DIVCF and risk of osteoporosis showed obviously beneficial effects (RR, 0.66; 95% CI, 0.48–0.92) (Figure 6).

### Meta-Analysis of DIVCF and BMD at the Lumbar Spine and Femoral Neck

**Figure 7:**
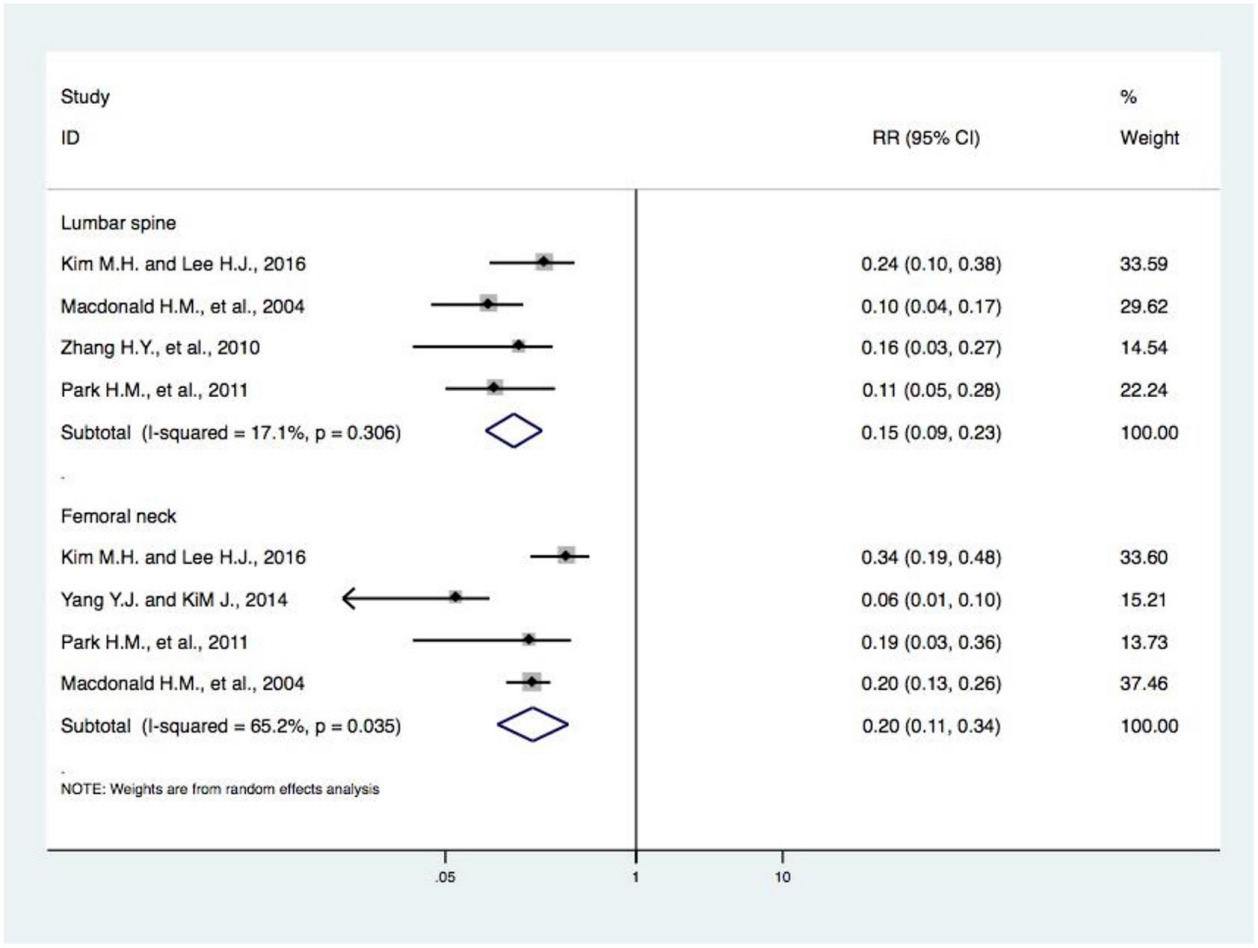
Forest plot of meta-analysis of DIVCF and BMD at the lumbar spine and at the femoral neck. The size of the boxes is proportional to the weight assigned to each study, and horizontal lines represent the 95% confidence interval. DIVCF, dietary intake of vitamin C-oriented foods; BMD, bone mineral density.

The risk of BMD loss at the lumbar spine was considered in four studies (17, 18, 21, 22). In our meta-analysis, we found that higher DIVCF was negatively associated with the risk of BMD loss at the lumbar spine (pooled *r*, 0.15; 95% CI, 0.09–0.23). Furthermore, four studies (17, 20–22) reported on DIVCF and the risk of BMD loss at the femoral neck. We found a significant negative association between them (pooled *r*, 0.20; 95% CI, 0.11–0.34) (Figure 7).

## Discussion

### Summary of Evidence

Our meta-analysis included 13 eligible articles (including 17 studies) with 19,484 subjects. The pooled RR of hip fracture for the highest DIVCF category vs. the lowest category was 0.66 (95% CI, 0.47–0.94), i.e., people with a high frequency of Vitamin C-oriented food intake had a 34% (95% CI, 6%−53%) lower prevalence of hip fracture. In our subgroup analyses stratified by study design, gender, and age, the negative associations were found to be statistically significant. Furthermore, the analysis of DIVCF and risk of osteoporosis (RR, 0.66; 95% CI, 0.48–0.92), BMD at the lumbar spine (pooled *r*, 0.15; 95% CI, 0.09–0.23), and BMD at the femoral neck (pooled *r*, 0.20; 95% CI, 0.11–0.34) showed high DIVCF has beneficial effects. Our meta-analysis indicates that DIVCF is negatively associated with the risk of hip fracture, osteoporosis, and BMD loss, suggesting that people should consume more Vitamin C to decrease the risk of hip fracture, osteoporosis, and BMD loss.

### Comparison of the Findings With Other Results in the Literature

In this paper, we carried out a meta-analysis of the possible protective effect of DIVCF against osteoporosis, fracture, and BMD loss. A similar article (27) was published in April 2018. The authors found no significant association between Vitamin C intake and the risk of hip fracture (RR, 0.74; 95% CI, 0.51–1.08). In our analysis, three more articles (with four studies) (16, 19, 20) were included, providing the chance to access documents that were published in Chinese. In this paper, we also construct a sound basis for further clinical research in this field (28). In addition to assessing the potential correlation between DIVCF and the risk of osteoporosis, we included meta-analyses on the effect of DIVCF on the risk of hip fracture and BMD loss. Finally, our pooled-effects analysis revealed a negative correlation between DIVCF and risk of hip fracture (RR, 0.66; 95% CI, 0.47–0.94); i.e., subjects with a higher intake of Vitamin C-oriented foods had a 34% (95% CI, 6%−53%) lower risk of hip fracture (Figure 2). Our results differ from those of Dr. Malmir, published in April 2018 (27). Due to the major heterogeneity among the studies, we performed subgroup analyses based on study type (i.e., cohort and case–control studies), gender, and age (middle-aged subjects, older subjects). Between DIVCF and risk of osteoporosis, a negative correlation was found (RR, 0.66; 95% CI, 0.48–0.92) (Figure 6). Also, we found that DIVCF was negatively associated with the risk of BMD loss at the lumbar spine (pooled *r*, 0.15; 95% CI, 0.09–0.23) and at the femoral neck (pooled *r*, 0.20; 95% CI, 0.11–0.34) (Figure 7).

Although a series of studies on Vitamin C and the risk of osteoporosis have been published, including controlled trials, case series, and case reports, to the best of our knowledge, no systematic meta-analyses have been published focusing on the correlation between DIVCF and osteoporosis, hip fracture, and BMD loss.

Several biological mechanisms may underlie the impact of DIVCF on the risk of osteoporosis. First, DIVCF includes not only Vitamin C but also Vitamin A, Vitamin B1, Vitamin B2, Vitamin B3, iron, and potassium intake. The positive effects of these vitamins and minerals on bone health have been well-established (29). Osteoporosis is a systemic skeletal disease, which is common in postmenopausal women and is characterized by an increased risk of bone fragility and a decrease in bone mass. Osteoporosis is often associated with metabolic syndrome (MBS), which involves oxidative stress and insulin resistance (30). Vitamin C-oriented foods are rich in several antioxidants, including Vitamin C, Vitamin K, and other phytochemicals. A high antioxidant consumption could contribute to lowering reactive oxygen species levels and enhancing the antioxidant status in animal models and human subjects (31, 32); this may slow down the progression of systemic oxidative damage resulting from overproduction of reactive oxygen species (33–35). Moreover, many phytochemicals can increase insulin production (36), and therefore potentially aid in the prevention of insulin resistance and hence MBS. Furthermore, DIVCF could protect against MBS by regulating the levels/activity of C-reactive protein and other inflammatory markers (37, 38). Higher DIVCF is correlated with reduced plasma concentrations of C-reactive protein (39, 40), and the latter might protect against MBS. Finally, people who eat many Vitamin C-oriented foods may consume much dietary fiber and little fat, decreasing the risk of MDS (41, 42). All the above mentioned factors are positively associated with BMD and negatively correlated with bone loss, thus reducing the risk of osteoporosis and fractures.

### Meaning of the Study for Possible Clinical Use

A positive link was found between DIVCF and a reduced risk of osteoporosis, fracture, and BMD loss. Several observations support the use of this information in clinical settings. First, our meta-analysis included a large number of subjects, reducing the sampling error to a large extent. Second, large efforts have been made to control the interference of possible confounding factors (such as age, BMI, physical activity level, and smoking status), resulting from the adjusted RR being fully extracted. Third, similar correlations were observed after performing subgroup analyses stratified by study type, gender, and age, confirming that the results are credible and robust.

### Limitation of the Review

Nevertheless, our study has several limitations. One of the key points is that only DIVCF was taken into account (and no dietary intake of other foods, such as fruit and vegetable), while many people with high DIVCF may in general eat a healthy diet, so an analysis of other healthy diets may give similar results. Therefore, it is necessary to verify that DIVCF is the active component of the diet. Also, it would be helpful to include intake of other dietary components (such as fruit and vegetable) for further study. Furthermore, Vitamin D plays a large role in the maintenance of BMD; its influence on the development of secondary hyperparathyroidism and osteomalacia, states where BMD is reduced reversibly, has been reported. Only a few studies in our meta-analysis adjusted for Vitamin D intake, though it is of great importance in the majority of nutritional studies (10, 12, 18–20). All the above mentioned factors could result in bias or inaccurate interpretations.

Another important point is that most of the articles we identified were cross-sectional or case–control studies; larger prospective cohort studies are warranted to verify the results. Although we have adjusted for most of the confounders the articles included, other confounders may still have existed. Moreover, the confounders that were adjusted for were different in each research; this may certainly have affected the results. Moreover, the approach of osteoporosis assessment differed between articles, which could influence the observed correlation. Because of the limited number of included articles, the effects of an inconsistent assessment approach on the strength of the correlation could not be further explored. Finally, due to the limited available data, we could not assess the dose–response association between DIVCF and the risk of osteoporosis.

Between-study heterogeneity is identified in most meta-analyses, and it is of importance to explore the sources of heterogeneity between studies. In our meta-analysis, there was high heterogeneity in the correlation between DIVCF and risk of osteoporosis. The factors contributing to such heterogeneity are complex. First, differences in the definition, classification, and assessment of DIVCF were found, which could result in heterogeneity. Second, the variables adjusted for (including age, BMI, energy intake, parathyroid hormone levels, 25-hydroxyvitamin D levels, smoking, alcohol intake, physical activity, supplement use, and oral contraceptive use) were not consistent between studies. In addition, because of the limited sample size, the evaluation of publication bias calls for further research.

## Conclusion

Our meta-analysis indicates that DIVCF is negatively associated with the risk of osteoporosis, hip fracture, and BMD loss and suggests that people should consume more DIVCF to reduce these risks.

## Author Contributions

JL and Z-PL conceived and designed the study. The literature searches, study selection, critical appraisal, data extraction, and contacting authors of included studies for additional information were carried out by L-FZ, M-HL, XX, Z-TG, DZ, J-LZ, J-TL, and J-KP. G-HL, JY, XW, H-YC, H-TH, QL, Y-HH, J-HL, S-RH, MW, and L-FZ conducted the interpretation and analysis of the relevant data. L-FZ, W-YY, DG, W-XL, QW, and JL drafted the paper. JL, A-HO, and L-FZ further revised the text. The final version of this article was rechecked and approved by L-FZ, M-HL, G-HL, M-HL, W-YY, XX, XW, JY, DG, H-YC, J-KP, H-TH, QL, Z-TG, Y-HH, DZ, J-LZ, S-RH, MW, J-TL, J-HL, W-XL, A-HO, QW, Z-PL, and JL.

### Conflict of Interest

The authors declare that the research was conducted in the absence of any commercial or financial relationships that could be construed as a potential conflict of interest.
