# Supplementary material for: Can Dietary Intake of Vitamin C-Oriented Foods Reduce the Risk of Osteoporosis, Fracture, and BMD Loss? Systematic Review With Meta-Analyses of Recent Studies
Source: Front Endocrinol (Lausanne). 2020 Feb 3;10:844. doi: 10.3389/fendo.2019.00844 (PMC7008177; doi:10.3389/fendo.2019.00844)

**Supplementary material**

**Supplementary Figure 1.** Funnel plots for the analysis of DIVCF and the risk of hip fracture: (a) study design; (b) age; (b) gender. RR, relative risk; SE, standard error; DIVCF, dietary intake of vitamin C-oriented foods. In the absence of publication bias, most points are symmetrically placed about the vertical line given by the pooled RRs. The reasonably symmetrical distribution suggests the absence of publication bias.

(a)


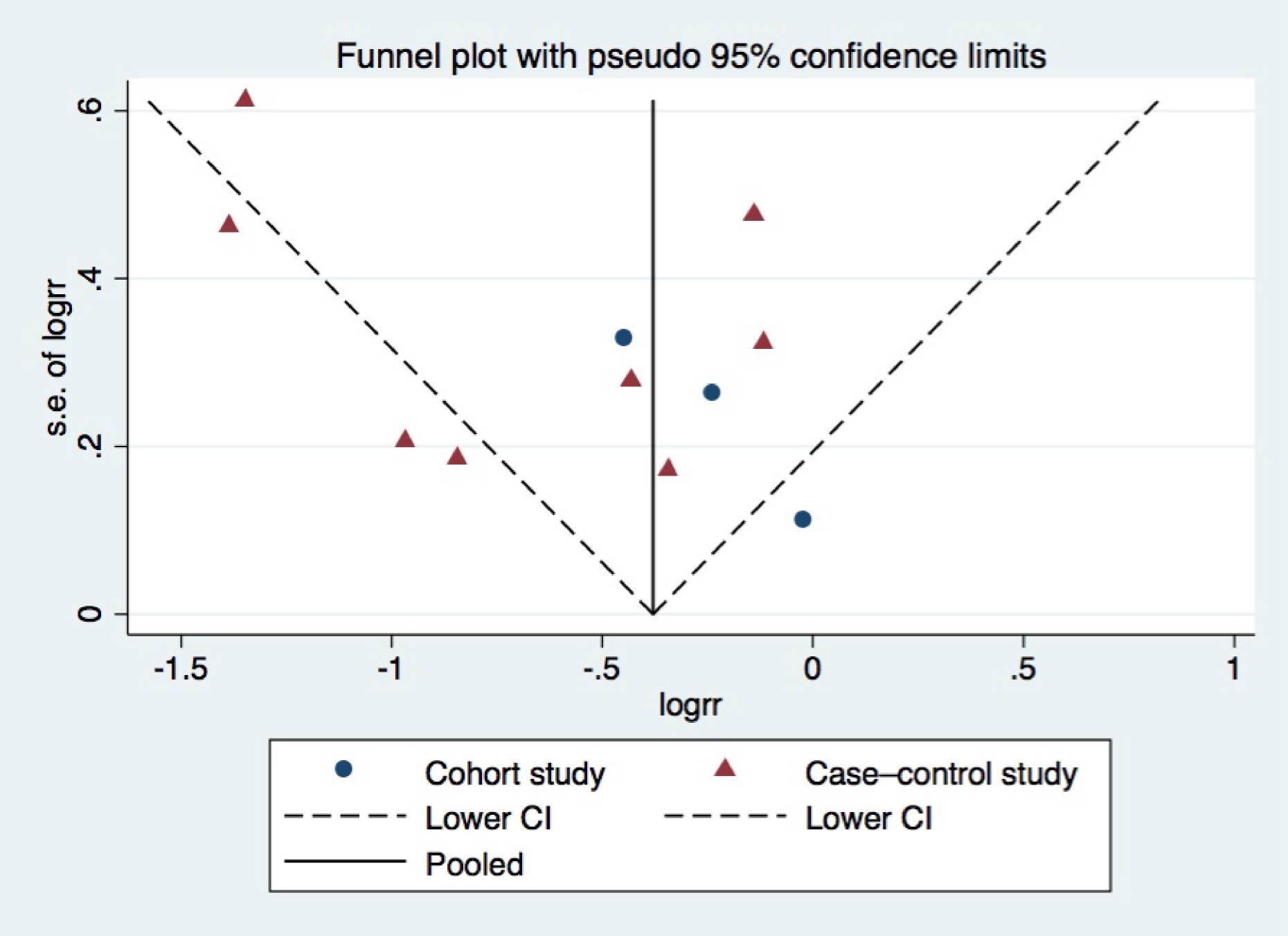


(b)


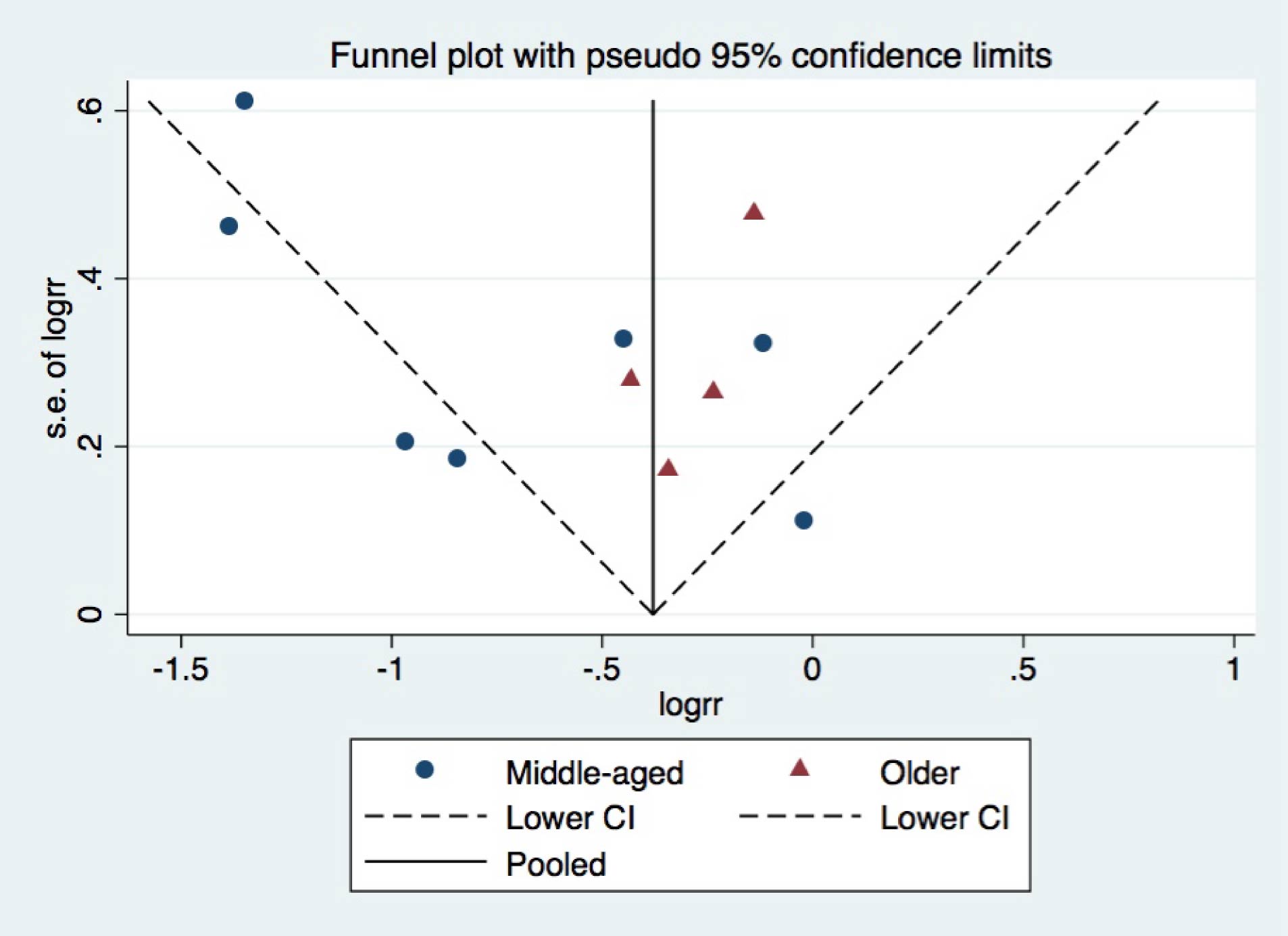


(c)


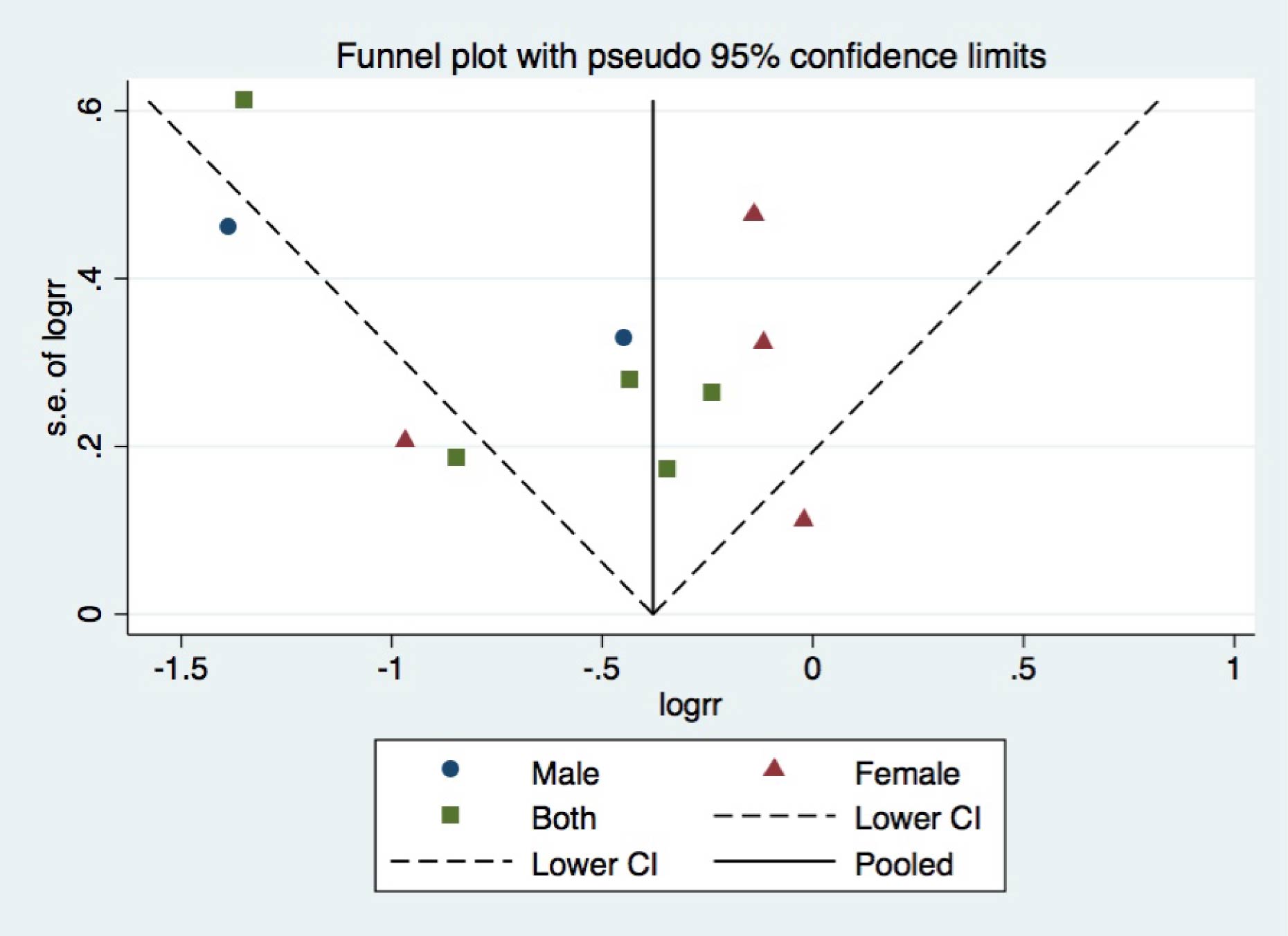

Supplement: Supplementary file 1 [file Table_3.DOC]
